# Limits of agricultural greenhouse gas calculators to predict soil N_2_O and CH_4_ fluxes in tropical agriculture

**DOI:** 10.1038/srep26279

**Published:** 2016-05-20

**Authors:** Meryl Richards, Ruth Metzel, Ngonidzashe Chirinda, Proyuth Ly, George Nyamadzawo, Quynh Duong Vu, Andreas de Neergaard, Myles Oelofse, Eva Wollenberg, Emma Keller, Daniella Malin, Jørgen E. Olesen, Jonathan Hillier, Todd S. Rosenstock

**Affiliations:** 1 CGIAR Research Program on Climate Change, Agriculture and Food Security, University of Copenhagen, Rolighedsvej 21, DK-1958 Frederiksberg C, Denmark; 2 Gund Institute, 617 Main Street, University of Vermont, Burlington VT 05405 USA; 3 Yale School of Management & Yale School of Forestry and Environmental Studies, 195 Prospect Street New Haven CT 06511 USA; 4 International Center for Tropical Agriculture, Km 17, Recta Cali-Palmira, Apartado Aéreo 6713 Cali, Colombia; 5 United Nations Development Programme, #53, Pasteur Street, Boeung Keng Kang P.O. Box 877, Phnom Penh, Cambodia; 6 Department of Soil Science and Agricultural Engineering, P.O.Box MP167 Mount Pleasant, University of Zimbabwe, Harare Zimbabwe; 7 Institute for Agricultural Environment, Vietnamese Academy of Agricultural Sciences, Vĩnh Quỳnh, Thanh Trì, Hanoi, Vietnam; 8 Department of Plant and Environmental Sciences, University of Copenhagen, 1871 Frederiksberg C, Denmark; 9 WWF-UK, The Living Planet Centre, Rufford House, Brewery Road, Woking, Surrey GU21 4LL, UK; 10 Sustainable Food Lab, 3 Linden Road, Hartland, VT 05048 USA; 11 Department of Agroecology, Blichers Allé 20, Aarhus University, Tjele 8830, Denmark; 12 School of Biological Sciences, University of Aberdeen, 23 St Machar Drive (G45), Aberdeen, AB24 3UU, UK; 13 World Agroforestry Centre, PO Box 30677-00100 Nairobi, Kenya

## Abstract

Demand for tools to rapidly assess greenhouse gas impacts from policy and technological change in the agricultural sector has catalyzed the development of ‘GHG calculators’— simple accounting approaches that use a mix of emission factors and empirical models to calculate GHG emissions with minimal input data. GHG calculators, however, rely on models calibrated from measurements conducted overwhelmingly under temperate, developed country conditions. Here we show that GHG calculators may poorly estimate emissions in tropical developing countries by comparing calculator predictions against measurements from Africa, Asia, and Latin America. Estimates based on GHG calculators were greater than measurements in 70% of the cases, exceeding twice the measured flux nearly half the time. For 41% of the comparisons, calculators incorrectly predicted whether emissions would increase or decrease with a change in management. These results raise concerns about applying GHG calculators to tropical farming systems and emphasize the need to broaden the scope of the underlying data.

Of the 189 countries that have submitted contributions^1^ to 2015 United Nations Framework Convention on Climate Change (UNFCCC) agreement in Paris, 119 include agriculture in their mitigation targets or actions. Over half of mitigation targets in non-Annex 1 countries included agriculture, which is unsurprising given that this sector represents the most significant fraction of national greenhouse gas (GHG) budgets^2^ and developing countries provide 70% of the potential for land-based mitigation^3^. Demand for tools to rapidly assess GHG impacts from policy and technological change has catalyzed the development of ‘GHG calculators’— simple accounting approaches that use a mix of emission factors and empirical models to calculate GHG emissions with minimal input data^4^.

GHG calculators provide an accessible approach for non-specialists to estimate GHG impacts from agriculture^5^ because they are inexpensive, rapid, and a relatively less knowledge-intensive option than other GHG quantification alternatives, such as *in-situ* measurement campaigns or process-based models^6^. The user-friendliness of GHG calculators has helped stimulate adoption among programs aiming to establish standards for quantifying and verifying emission reductions and companies committed to reduce environmental impacts in their supply chains^7^,^8^. However, the ability of GHG calculators to predict soil emissions is uncertain, given GHG calculators rely on models calibrated from measurements conducted overwhelmingly under temperate, developed country conditions^9^.

We compiled GHG emissions data from field experiments in tropical smallholder systems and compared them with predicted emissions from two of the most commonly used GHG calculators, EX-ACT^10^ and Cool Farm Tool (CFT)^5^. We compared measured fluxes and C stock changes derived from 51 experimental treatments from nine studies in Africa, Asia, and Latin America to the predicted fluxes and stock changes estimated by CFT and EX-ACT. Experimental sites spanned a diversity of emission drivers including low/high input systems and upland/flooded conditions (Table 1). Both calculators use ‘activity data’—data on the magnitude of human activity that generates emissions or removals—combined with empirical models and emission factors (IPCC Tier 1) to estimate fluxes of nitrous oxide (N_2_O) from soils, carbon (C) sequestration in above and belowground biomass, and methane (CH_4_) production from flooded rice cultivation and other farm activities (Table 2).

Many GHG calculators allow users to input locally derived Tier 2 emissions factors instead of applying Tier 1 global defaults. However, GHG calculators are virtually always applied using Tier 1 emission factors due to the lack of available data for many regions and emissions sources. It should be noted that Tier 1 methods were developed for national scale accounting and were to be used for emission sources relatively inconsequential to overall budget (e.g., less than 5% of the total), and therefore would not be expected to provide accurate field-scale emissions estimates in either temperate or tropical systems. However, Tier 1 factors are used as the basis for many GHG impact assessments using these calculators (for example see Chakrabarti^11^) as they are the only option available. We, therefore, evaluated the calculators as they primarily are being applied, using Tier 1 at field-scale.

There are three differences between the calculators that were relevant to our study. First, CFT uses an exponential model to estimate N_2_O that is sensitive to soil and climate variables and includes background soil emissions and emissions from crop residues^12^,^13^, whereas EX-ACT uses the default IPCC Tier 1 emission factor (1% of nitrogen applied to soil^14^). Both the model and emission factor were calculated from the same data set^12^. Second, the tools differ in how they treat crop residues. In CFT, users input crop yields and residue treatment and the tool estimates the nitrogen content of the residues and the resulting N_2_O emissions. In EX-ACT, N_2_O emissions from residue are not calculated unless the tool user enters the nitrogen content of the residues as a fertilizer input. Third, CFT accounts for multiple organic amendments (e.g. rice straw residues and manure) in estimating CH_4_ emissions from flooded rice, while EX-ACT requires the user to choose either rice straw or another organic amendment. An additional distinction between the calculators is that CFT is a commodity-focused calculator and must be run multiple times to account for multiple crops or farms, while EX-ACT can be used at the landscape scale. However, because we examined field-level practices of monocultures, this difference was not relevant to our study.

## Results

**Figure 1:**
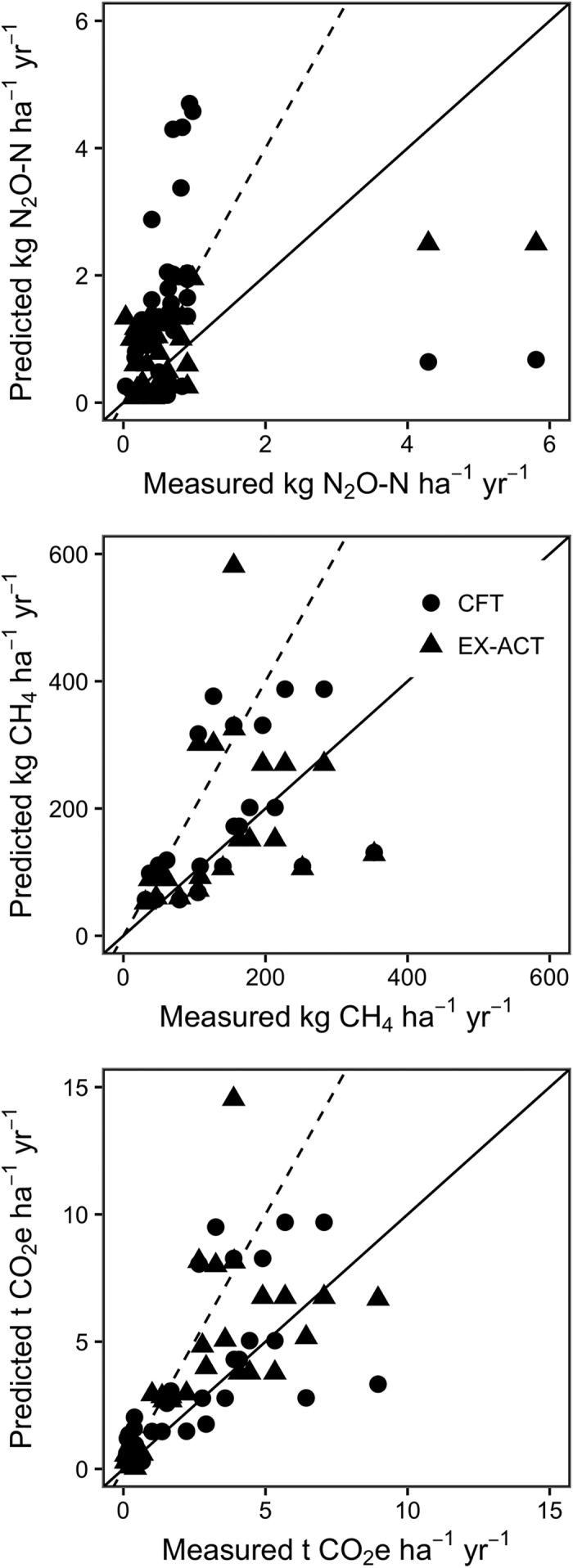
Comparison between measured and calculator-predicted soil fluxes for N_2_O, CH_4_, and the net balance (CO_2_e). The solid line is a 1:1 line; data points above this line represent an over-estimation of GHG emissions by the calculator. The dashed line is a 1:2 line; data points above this line represent an overestimation by a factor of 2 or more.

Our analysis shows that CFT consistently overestimated net GHG emissions from the agricultural systems represented in this sample (Fig. 1), while EX-ACT over-estimated and under-estimated emissions in near-equal proportions. In an average of 70% of cases (55% of EX-ACT and 77% of CFT cases), the calculator-predicted GHG emissions were greater than the measured emissions. In 41% of cases (29% of EX-ACT and 47% of CFT cases), predicted emissions exceed twice those obtained through field measurements (Fig. 1). Calculator estimates were within the 95% confidence interval of the measured value in just 19% of cases. The overestimation by CFT was due primarily to N_2_O estimates; the median ratio of predicted to measured values was 2.1 (interquartile range = 0.7-4.0) for N_2_O and 1.3 (interquartile range = 0.8-2.0) for CH_4_. EX-ACT estimates of N_2_O tended to be lower in general because EX-ACT does not include emissions from crop residues in its calculations, whereas CFT does calculate N_2_O emissions from residue. Where residue inputs are high, especially from nitrogen-fixing crops, EX-ACT may therefore underestimate N_2_O emissions. The calculators were not biased towards overestimation with CH_4_, and in fact underestimated CH_4_ emissions in several cases, particularly those involving manure and compost inputs to rice. Calculator estimates were within the 95% confidence interval of the measured value in 23% of cases for CH_4_ and only 5% of cases for N_2_O, with only a 2% difference between the calculators. Slightly better agreement of CH_4_ estimates may be attributed to the larger existing body of literature on CH_4_ fluxes and the inherent difficulty in predicting N_2_O fluxes due to the complex drivers of the flux and its temporal and spatial variability^15^. In systems where multiple sources and sinks (N_2_O and CH_4_ or C storage) were included in the calculation, the median ratio of predicted to measured GHG balances was 1.7 (interquartile range = 0.8-2.7).

Despite the CFT’s tendency to overestimate N_2_O, the calculators did not otherwise differ statistically in terms of accuracy. Differences between measured and calculator-estimated N_2_O emissions were not significantly different between CFT and EX-ACT (Wilcoxon signed ranks test, Z = −0.169, *p *= 0.866). This finding contradicts our expectation that the exponential model employed by the CFT would be more accurate based on recent meta-analysis showing an exponential response of N_2_O emissions to N inputs to soils^16^. The overestimation of the exponential model in our analysis, however, likely reflects the underlying data upon which it is based rather than a contradiction of an exponential response per se. The model coefficients in the CFT were developed using data primarily from temperate climates with N application rates between 100 and 250 kg N/ha; less than 5% of the studies were conducted in the tropics and sub-tropics, and few included a zero N control^12^,^16^. This model may represent high input systems on fertile soils in developing countries (e.g., China) well^17^ but perhaps be less relevant for low input systems characteristic of many other regions^18^. N inputs in the treatments included in our analysis ranged from 0 to 250 kg N/ha, with a mean of 97 N kg/ha, and given the evidence on non-linearity of N_2_O emissions to input, extrapolation is not trivial. Attempts to improve both the exponential model used in CFT and the IPCC emission factor used in EX-ACT, both of which are based on the same data set, have so far been limited by a lack of empirical studies in tropical climates^12^,^16^. Studies with multiple N input levels would help determine the inflection point of the exponential response and improve the model’s ability to predict N_2_O emissions^16^.

No statistical inferences can be drawn about the accuracy of soil C estimates as only six data points in our data set included measurements of soil C sequestration. However, the response of soil C stocks to management changes is poorly quantified in general^19^. This is particularly true in tropical countries where there are few long-term studies of C stock changes, which is reflected in the higher uncertainties associated with IPCC soil C stock change factors relative to temperate regions^14^. While our dataset is too limited to draw grand conclusions about C stock changes, this is true of the entire body of scientific literature at this time. There is an acute need for more quantitative studies across diverse soil types and management practices to establish relative sequestration potential and C saturation levels^20^.

**Figure 2:**
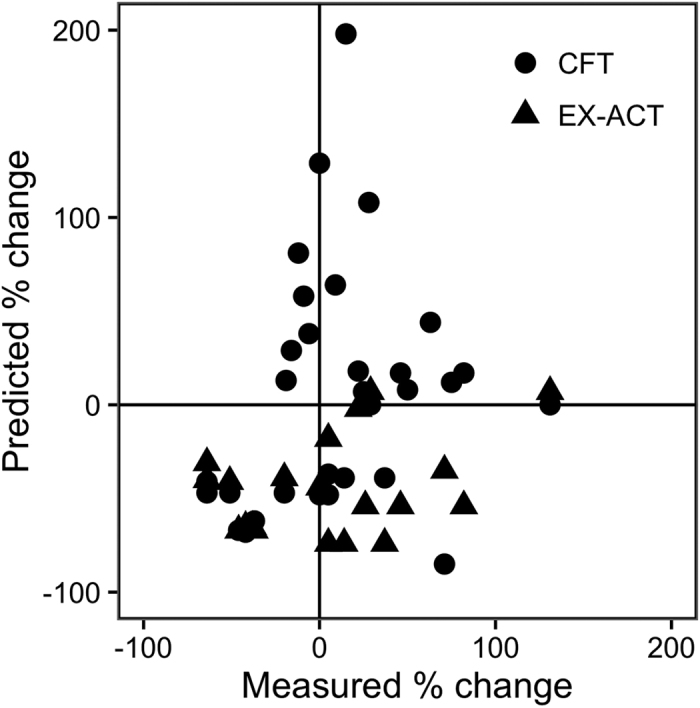
Change in GHG balance between control and alternative management practices (e.g. continuous flooding vs. multiple drainage in rice). Points in the upper right and lower left quadrants represent cases where the calculator predicted the same direction of change as observed in the field study. Points in the lower right and upper left quadrants represent cases where the calculator predicted the opposite direction of change as observed in the field study.

Though GHG calculators can be used to provide estimates of the magnitude of emissions from a given system, often companies, project managers, and farmers use them to anticipate or monitor the effects of mitigation practices^6^. The most relevant question about the utility of such calculators is therefore whether they can predict the relative change in GHG balance associated with a change in management. To analyze this, we calculated the percent change in GHG balance between control and alternative management practices in studies within our data set that included multiple treatments (n > 1 in Table 1). We then compared the calculator-predicted changes with the measured changes. Of the 33 comparisons, CFT and EX-ACT correctly predicted the direction of change for 65% and 50% of cases, respectively (Fig. 2). Calculator predictions and measurements contradicted each other for 41% of cases, indicating that GHG balance changes with the management change were negative when measured but positive when estimated by the calculator, or vice versa.

A Wilcoxon paired signed-rank test showed that changes in GHG balance associated with a change in management were significantly different between the calculator estimations and the measured values for both N_2_O (*Z *= −3.323, *p *= 0.001) and CH_4_ (*Z *= 20, *p *= 0.000), meaning that the calculators were unable to accurately predict changes in emissions with changes in management.

The calculators were less able to predict directional changes when a combination of practices were used (in particular a change in water management and organic inputs in flooded rice) or where N_2_O emissions were so low that differences were barely distinguishable, such as maize cultivation without fertilizer. On the other hand, the calculators predicted the direction of change correctly for practice changes with relatively well-understood effects on emissions, such as differing levels of mineral nitrogen fertilizer or intermittent drainage of flooded rice with no change in organic inputs.

## Discussion

Our analysis illustrates the challenges of using GHG calculators to predict emissions both in absolute and relative terms. Other GHG calculators use similar approaches as those evaluated here: IPCC Tier 1 emission factors or empirical models parameterized by data collected in temperate climates (Table 2). For example, the IPCC Tier 1 emissions factor for N_2_O fluxes resulting from fertilizer application was developed from a meta-analysis of 1891 studies, yet fewer than 10% were from cultivated soils in tropical countries^12^. The congruence in methods and underlying data among the CFT, EX-ACT and other calculators (Table 2) suggests that our findings can also be expected for other GHG calculators using Tier 1 methods.

These findings have important implications for when and how GHG calculators can be used. Because GHG calculators do not produce absolute or relatively accurate results with much certainty, application of these calculators for GHG accounting or monitoring, verification and reporting (MRV) purposes cannot be performed with reliable results in tropical systems with low inputs or interacting practices. In these systems, the calculators may provide spurious estimates of GHG fluxes. More importantly, in comparing mitigation options, the calculator user risks incorrectly predicting emissions reduction potentials or predicting emissions changes where none may occur.

The CFT’s tendency to overestimate emissions may be seen as unproblematic and in fact preferable to underestimation for applications such as life cycle analysis where a “worst-case” situation is often assumed. In our data set, it was cases involving application of organic inputs to rice, and especially combinations of changes in water management and organic inputs, where both tools underestimated emissions and incorrectly predicted emissions reductions. Users of GHG calculators and Tier 1 estimates in general should be aware of these limitations if using these tools to estimate an “upper bound” of emissions.

A limitation of this study was the generally high uncertainty associated with measurement of agricultural emissions^21^. In low-emissions environments, standard errors associated with soil GHG flux measurements may be of nearly the same magnitude as the fluxes themselves, which is reflected in the high standard deviations reported in this studies from which we have drawn data. Additionally, the frequency and duration of the field measurements highly influence cumulated measured fluxes, especially N_2_O^22^,^23^. The data included in this paper were drawn from studies that measured emissions at a weekly frequency and for an entire growing season, with the exception of Nyamadzawo *et al*.^24^ who measured every other week. However, given the high temporal and spatial variability of N_2_O fluxes, even measurements at a high temporal resolution may misrepresent true cumulative emissions. This highlights the importance of uncertainty estimates in output from GHG calculators. While EX-ACT provides an uncertainty estimate based on the aggregated uncertainty of the emission factors used, CFT does not. Without some indication of uncertainty, GHG calculators give the false impression of precision and fail to provide consumers of the information crucial understanding of the risks involved in their actions. Greater attention needs to be made to provide measures of uncertainty as well as helping policy makers and program developers interpret results and act despite the uncertainty.

The inaccuracy of GHG calculators is reflective not of the construction of the calculators themselves, but of the underlying models and emission factors from which they have been constructed. In fact, GHG calculators may perform only marginally better in temperate areas when Tier 1 emission factors are used, given the substantial uncertainty associated with these factors. For example, Stehfest and Bouwman report a confidence interval of −51% to +107% for N_2_O estimates^23^. In temperate areas, however, more locally specific emission factors are often available. Improving the accuracy of GHG calculators in the tropics depends on calibration of the underlying factors and models to the environmental conditions and systems common in agriculture in tropical developing countries. In the short term, IPCC-approved Tier 2 emission factors based on currently available data may help provide a more reasonable picture of both current fluxes and mitigation potential. N_2_O estimates would be improved by emission factors adjusted by nitrogen input levels and, perhaps, by additional factors such as nitrogen source, placement, timing as well as soil moisture, plant composition, or soil fauna^16^,^25^,^26^. Calibration of empirical models, such as the N_2_O model used in the CFT^12^,^25^ for conditions more representative of tropical developing countries would likely also improve estimates^16^. Given the large variation in emission rates, both below 1% of nitrogen applied^23^ and above 4% of nitrogen input derived from top-down approaches^27^ in tropical and temperate systems, there is a significant need to better understand the mechanisms involved in GHG evolution from agricultural soils in a way that can be used in simple calculators.

Our current ability improve our understanding of mechanisms driving GHG emissions under tropical conditions is data-limited. For example, there are few published studies from Africa on the response function of N_2_O to N inputs^18^, and few long-term (>10 years) studies of soil carbon sequestration in the tropics. Data characterizing enteric CH_4_ emissions from livestock systems are another critical gap; we were unable even to compare calculator-produced estimates with measured emissions due to the lack of published studies with field measurements from tropical developing countries. Therefore, in the long-term, there is a need for additional data to revise and recalibrate these calculators for tropical systems.

## Methods

We compiled GHG emissions data from studies of tropical smallholder systems. In total, we analyzed 51 data points from nine studies located in seven smallholder cropping systems in eight countries. Each data point or “case” represents a unique experimental treatment in one of the studies included in this analysis. These studies include a diversity of key predictors including low to high input systems, upland to flooded conditions, and a range of continents (Table 2). We supplemented data provided in the publications with additional agricultural management details provided by authors to improve the detail and accuracy of the data input into the calculators.

Comparisons were made between the measured emissions and sequestration rates and the estimation output from the Cool Farm Tool and EX-ACT Tool. Because much of the available measured data generally did not calculate whole-farm GHG balances, we used only selected modules from the GHG calculators for comparison. We did not, for example, include emissions from fertilizer production, as these emissions were not accounted for in the field measurement studies. Furthermore, because EX-ACT does not estimate background N_2_O emissions or N_2_O emissions from residue, we did not include EX-ACT estimates for treatments in which no nitrogen fertilizer (synthetic or organic) was applied. EX-ACT can in theory calculate emissions from crop residues as well, if the nitrogen content of residues is entered under “other nitrogen fertilizers”; for this study we did not do so, on the basis that most GHG calculator users would (a) not consider crop residues “fertilizers” and (b) not know the precise quantity and N content of the crop residues. Detailed information on the data input into the calculators can be found as Supplementary Tables S1 and S2.

EX-ACT and CFT were selected because they represent user-friendly GHG calculators that are among the most widely applied in the private sectors and development organizations. Both calculators use a mix of simple emission factor approaches (IPCC Tier 1) and empirical models to calculate net GHG emissions without the need for data input beyond basic climate, soil, and farm management information. EX-ACT allows the user to enter site-specific emission factors (IPCC Tier 2) where available, but we used the default values in our comparison for comparability between calculators and in order to reflect how the calculators would be used in the data-limited situations most common in developing countries.

We used the ratio of the predicted estimate to the measured estimates as an indicator of accuracy. To compare calculators and GHG estimations (CH_4_ and N_2_O), we used the two-tailed Mann-Whitney *U* test. To examine how well the calculators predicted changes in GHG balance between control and alternative management treatments, we used the two-tailed Wilcoxon paired signed-ranks test.

## Additional Information

**How to cite this article**: Richards, M. *et al*. Limits of agricultural greenhouse gas calculators to predict soil N_2_O and CH_4_ fluxes in tropical agriculture. *Sci. Rep.* **6**, 26279; doi: 10.1038/srep26279 (2016).

## Supplementary Material

Supplementary Information

## Figures and Tables

**Table 1:** Experimental results used in this study (*n *= 51).

**Country**	**City/town**	**Coordinates**	**Climate**	**Crop(s)**	**Sources/sinks**	**n**	**Reference**
Cambodia	Trapeang Thom Khang Cheung	11°3′N, 104°34′E	Tropical	Rice (*Oryza sativa L.*)	CH_4_	8	28
China	Nanjing	31°52′N, 118°50′E	Tropical	Rice (*Oryza sativa L.*)	CH_4_, N_2_O	6	29
Costa Rica	San Pedro	10°2′N, 84°8′W	Tropical	Coffee (*Coffea arabica*)	Biomass C, soil C, N_2_O	2	30
Kenya	Kaptumo	0°4′N, 35°4′E	Tropical	Napier grass (*Pennisetum purpureum*)	N_2_O	1	Rosenstock *et al*. forthcoming
Kenya	Kaptumo	0°4′N, 35°4′E	Tropical	Tea (*Camellia sinensis*)	N_2_O	2	Rosenstock *et al*. forthcoming
Kenya	Kaptumo	0°4′N, 35°4′E	Tropical	Vegetables (*Solanum tuberosum L., Brassica oleracea*)	N_2_O	2	Rosenstock *et al*. forthcoming
Kenya	Maseno	0°0′N, 34°35′E	Tropical	Maize (*Zea mays*)	N_2_O	5	31
Mexico	Texcoco	19°19′N, 98°30′ W	Subtropical	Maize (*Zea mays*)	Soil C, N_2_O	4	32
Tanzania	Kolero	37°48′ E, 7°15′ S	Tropical	Cassava (*Manihot esculenta*)	N_2_O	1	Rosenstock *et al*. forthcoming
Tanzania	Kolero	37°48′ E, 7°15′ S	Tropical	Maize (Zea mays)	N_2_O	1	Rosenstock *et al*. forthcoming
Tanzania	Kolero	37°48′ E, 7°15′ S	Tropical	Maize (*Zea mays*)	N_2_O	5	33
Vietnam	Hanoi	21°20′N, 106°1′E	Tropical	Rice (*Oryza sativa L*.)	N_2_O, CH_4_	8	34
Zimbabwe	Domboshawa	17°42′ S, 31°0′ E	Tropical	Maize (*Zea mays*)	N_2_O	6	24

**Table 2:** Calculation methods used in four GHG calculators used in developing countries.

**Calculator name**	**Scale**	**Background soil N**_**2**_**0**	**N**_**2**_**0 from fertilizers**	**N**_**2**_**O from crop residue management**	**CH**_**4**_ **from rice cultivation**	**Soil C stock changes**	**Biomass C stock changes within a land use category**
Cool Farm Tool	Farm	Multivariable empirical model^c^	Multivariable empirical model^c^	Single emission factor^a^	Multiple emission factors^a^,^d^	Multiple emission factors^a^,^e^	Allometric equations^a^
EX-ACT^g^	Landscape	Not included	Single emission factor^a^	Not included unless entered as a fertilizer	Multiple emission factors^a^,^d^	Multiple emission factors^a^,^b^	Not included
USAID AFOLU Carbon Calculators Cropland Management Tool	Landscape	Not included	Single emission factor^a^	Not included	Multiple emission factors^a^,^d^	Multiple emission factors^a^,^b^	Not included
Carbon Benefits Project simple assessment tool^h^	Landscape	Not included	Single emission factor^a^	Single emission factor^1^	Multiple emission factors^a^,^d^	Multiple emission factors^a^,^b^,^f^	Allometric equations^a^

^a^IPCC, 2006.

^b^Ogle *et al*., 2005.

^c^Bouwman *et al*., 2002; Stehfest and Bouwman, 2006.

^d^Yan *et al*. 2005.

^e^Smith *et al*. 1997.

^f^Batjes *et al*. 2011.

^g^User can add Tier 2 EFs. Cannot include more than one organic soil amendment in rice CH_4_ calculation.

^h^CBP simple assessment uses IPCC (2006) EFs; detailed assessment can utilize locally-developed Tier 2 Efs for C stock changes; dynamic modeling option uses Century process-based model.
