# Supplementary material for: Limits of agricultural greenhouse gas calculators to predict soil N2O and CH4 fluxes in tropical agriculture
Source: Sci Rep. 2016 May 20;6:26279. doi: 10.1038/srep26279 (PMC4873796; doi:10.1038/srep26279)
Supplement: Supplementary Information [file srep26279-s1.pdf]

Limits of agricultural greenhouse gas calculators to predict soil N_2O and CH_4 fluxes in tropical agriculture - Supplementary Information

Meryl Richards, Ruth Metzel, Ngonidzashe Chirinda, Proyuth Ly, George Nyamadzawo, Quynh Duong Vu, Andreas de Neergaard, Myles Oelofse , Eva Wollenberg, Emma Keller, Daniella Malin, Jørgen E Olesen, Jonathan Hillier, Todd S Rosenstock*

Corresponding author: t.rosenstock@cgiar.org

The following four tables detail the parameters used to estimate greenhouse gas (GHG) fluxes with the two GHG calculators: Cool Farm Tool (CFT) (Tables S1a & S2a) and EX-ACT (Tables S1b & S2b). Tables S1a & S1b relate to the same experiments conducted in upland cropping systems but differ in the GHG calculator applied, CFT and EX-ACT, respectively. The experiments detailed in Tables S2a & S2b relate to the same experiments conducted in flooded rice systems and relate to CFT and EX-ACT, respectively. Measured emissions for the site are listed next to GHG calculator estimates. Standard errors for measured emissions were converted to CO_2e, calculated from standard deviations, or extrapolated from graphical data where necessary.

Table S1a Description of upland crop experimental treatments as entered into CFT with calculator-estimated and measured GHG emissions

Treatments				Cool Farm Tool: Farm Settings		Cool Farm Tool: General		Cool Farm Tool: Growing area					Cool Farm Tool: Field treatment				Cool Farm Tool: Management			Cool Farm Tool: Estimate ($tCO2e\ ha^{-1}\ yr^{-1}$)			Measured: Estimate ($tCO2e\ ha^{-1}\ yr^{-1}$)			
Country	Crop	Treatment	Reference	Climate	Average temperature	Product	Crop type	Soil texture	% SOM	Soil moisture	Soil drainage	Soil pH	Fertilizer 1	Fertilizer 2	Amount of residue	Residue treatment	Management Changes	Tree/bush species 1	Tree/bush species 2	N_2O	CO_2	Total	N_2O	CO_2	Total	SE*
Costa Rica	Coffee	Full sun	Hergoualc'h et al. 2012	Tropical	21	Coffee	Coffee	Medium	5.23	Moist	Good	pH <= 5.5	180 kg/ha N urea, broadcast	70 kg/ha N ammonium nitrate, broadcast	3 t/ha	Left on field		Coffee (arabica) 5000 trees/ha		0.30	-0.12	0.18	2.01	-5.83	-3.82	1.98
Costa Rica	Coffee	Shade/agroforestry	Hergoualc'h et al. 2012	Tropical	21	Coffee	Coffee	Medium	4.9	Moist	Good	pH <= 5.5	180 kg/ha N urea, broadcast	70 kg/ha N ammonium nitrate, broadcast	4.9 t/ha	Left on field		Coffee (arabica) 4722 trees/ha	Shade (Cordia alliodora, etc.) 278 trees/ha	0.32	-10.24	-9.92	2.72	-17.31	-14.59	2.20
Kenya	Napier grass	Manure	Rosenstock et al. forthcoming	Tropical	18	Napier grass	Perennial grass	Medium	6.42	Moist	Good	5.5 < pH <= 7.3	12.5 t/ha cattle farmyard manure, broadcast		n/a	Exported off farm				0.23		0.23	0.23		0.23	
Kenya	Tea	Farm 1	Rosenstock et al. forthcoming	Tropical	18	Tea	Tea	Coarse	5.88	Moist	Good	pH <= 5.5	144 kg/ha N compound NPK		100 kg/acre	Left on field				0.94		0.94	0.22		0.22	
Kenya	Tea	Farm 2	Rosenstock et al. forthcoming	Tropical	18	Tea	Tea	Medium	5.06	Moist	Good	pH <= 5.5	141 kg/ha N compound NPK, broadcast		100 kg/acre	Left on field				0.60		0.60	0.11		0.11	
Mexico	Maize	Conventional till, residues removed	Dendooven et al. 2012	Tropical	14	Maize	Maize	Fine	2.10	Dry	Poor	5.5 < pH <= 7.3	150 kg/ha N urea, incorporated		n/a	Exported off farm				2.03	0.00	2.03	0.39	0.00	0.39	
Mexico	Maize	Conventional tillage, residues retained	Dendooven et al. 2013	Tropical	14	Maize	Maize	Fine	2.10	Dry	Poor	5.5 < pH <= 7.3	150 kg/ha N urea, incorporated		3.77 t/ha (CFT calculated from yield)	Left on field	Started incorporating residue 19 years ago			2.14	-0.94	1.20	0.46	-0.32	0.14	
Mexico	Maize	Zero tillage, residues removed	Dendooven et al. 2014	Tropical	14	Maize	Maize	Fine	2.10	Dry	Poor	5.5 < pH <= 7.3	150 kg/ha N urea, broadcast			Exported off farm	Changed to no-till 19 years ago			2.01	-1.71	0.30	0.33	0.34	0.66	
Mexico	Maize	Zero tillage, residues retained	Dendooven et al. 2015	Tropical	14	Maize	Maize	Fine	2.10	Dry	Poor	5.5 < pH <= 7.3	150 kg/ha N urea, broadcast		5.40 t/ha (CFT calculated from yield)	Left on field	Changed to no-till 19 years ago; started incorporating residue 19 years ago			2.20	-3.07	-0.87	0.44	-5.11	-4.68	
Zimbabwe	Maize	No inputs	Nyamadzawo et al. 2014	Tropical	18	Maize	Maize	Coarse	1.03	Moist	Good	pH <= 5.5				Exported off farm				0.29		0.29	0.10		0.10	0.01
Zimbabwe	Maize	Low mineral N	Nyamadzawo et al. 2014	Tropical	18	Maize	Maize	Coarse	1.03	Moist	Good	pH <= 5.5	60 kg/ha N ammonium nitrate, broadcast			Exported off farm				0.42		0.42	0.15		0.15	0.02
Zimbabwe	Maize	High mineral N	Nyamadzawo et al. 2014	Tropical	18	Maize	Maize	Coarse	1.03	Moist	Good	pH <= 5.5	120 kg/ha N ammonium nitrate, broadcast			Exported off farm				0.61		0.61	0.12		0.12	0.01
Zimbabwe	Maize	Mixed fertilization	Nyamadzawo et al. 2014	Tropical	18	Maize	Maize	Coarse	1.03	Moist	Good	pH <= 5.5	60 kg/ha N ammonium nitrate, broadcast	60 kg N/ha cattle manure, broadcast		Exported off farm				0.48		0.48	0.10		0.10	0.01
Zimbabwe	Maize	Low organic N	Nyamadzawo et al. 2014	Tropical	18	Maize	Maize	Coarse	1.03	Moist	Good	pH <= 5.5	60 kg/ha N cattle manure, broadcast			Exported off farm				0.33		0.33	0.08		0.08	0.02
Zimbabwe	Maize	High organic N	Nyamadzawo et al. 2014	Tropical	18	Maize	Maize	Coarse	1.03	Moist	Good	pH <= 5.5	120 kg/ha N cattle manure, broadcast			Exported off farm				0.38		0.38	0.08		0.08	0.02
Kenya	Vegetables	Manure and mineral fertilizer	Rosenstock et al. forthcoming	Tropical	18	Vegetables	Vegetables	Medium	5.97	Moist	Good	5.5 < pH <= 7.3	185.4 kg/ha cattle manure, incorporated	56.25 kg/ha diammonium phosphate, incorporated	0.71 t/ha (CFT calculated from yield)	Left on field				0.91		0.91	0.26		0.26	
Kenya	Potatoes	Manure	Rosenstock et al. forthcoming	Tropical	18	Potatoes	Potatoes	Medium	7.09	Moist	Good	5.5 < pH <= 7.3	4 t/ha cattle manure, broadcast		1.37 t/ha (CFT default calculated from yield)	Left on field				0.78		0.78	0.26		0.26	
Tanzania	Maize	Farmer practice (no inputs)	Rosenstock et al. forthcoming	Tropical	24	Maize	Maize	Coarse	2.83	Moist	Good	5.5 < pH <= 7.3			1.72 t/ha (CFT default calculated from yield)	Left on field				0.95		0.95	0.10		0.10	0.61
Tanzania	Maize	Conventional tillag, no inputs	Kimaro et al. 2015	Tropical	24	Maize	Maize	Coarse	4.64	Moist	Good	5.5 < pH <= 7.3				Exported off farm				0.59		0.59	0.08		0.08	0.02
Tanzania	Maize	No-till, leguminous intercrop, residue mulch	Kimaro et al. 2015	Tropical	24	Maize	Maize	Coarse	4.64	Moist	Good	5.5 < pH <= 7.3			1.34 t/ha	Left on field				0.64		0.64	0.08		0.08	0.05
Tanzania	Maize	No-till, mineral fertilizer, residue mulch	Kimaro et al. 2015	Tropical	24	Maize	Maize	Coarse	4.64	Moist	Good	5.5 < pH <= 7.3	100 kg/ha N urea		1.47 t/ha	Left on field				1.35		1.35	0.10		0.10	0.06
Tanzania	Maize	Heavy mulching in rows	Kimaro et al. 2015	Tropical	24	Maize	Maize	Coarse	4.64	Moist	Good	5.5 < pH <= 7.3			1.22 t/ha	Left on field				0.63		0.63	0.07		0.07	0.06
Tanzania	Maize	No-till, residue mulch, interplanting of leguminous trees	Kimaro et al. 2015	Tropical	24	Maize	Maize	Coarse	4.64	Moist	Good	5.5 < pH <= 7.3			1.94 t/ha	Left on field				0.66		0.66	0.10		0.10	0.07
Tanzania	Cassava	No inputs	Rosenstock et al. forthcoming	Tropical	24	Cassava	Other tuber crop	Coarse	1.67	Moist	Good	5.5 < pH <= 7.3			1.23 t/ha (CFT calculated from yield)	Left on field				0.64		0.64	0.13		0.13	0.22
Kenya	Maize	No N	Hickman et al. 2014	Tropical	24	Maize	Maize	Fine	3.09	Good	Good	pH <= 5.5				Exported off farm				0.53		0.53	0.33		0.33	0.04
Kenya	Maize	50 kg/ha N	Hickman et al. 2014	Tropical	24	Maize	Maize	Fine	3.09	Good	Good	pH <= 5.5	16.70 kg/ha N diammonium phosphate	33.3 kg/ha N urea		Exported off farm				0.73		0.73	0.31		0.31	0.05
Kenya	Maize	75 kg/ha N	Hickman et al. 2014	Tropical	24	Maize	Maize	Fine	3.09	Good	Good	pH <= 5.5	25.00 kg/ha N diammonium phosphate	50 kg/ha N urea		Exported off farm				0.84		0.84	0.30		0.30	0.05
Kenya	Maize	100 kg/ha N	Hickman et al. 2014	Tropical	24	Maize	Maize	Fine	3.09	Good	Good	pH <= 5.5	33.30 kg/ha N diammonium phosphate	66.7 kg/ha N urea		Exported off farm				0.96		0.96	0.29		0.29	0.04
Kenya	Maize	200 kg/ha N	Hickman et al. 2014	Tropical	24	Maize	Maize	Fine	3.09	Good	Good	pH <= 5.5	66.70 kg/ha N diammonium phosphate	133.3 kg/ha N urea		Exported off farm				1.58		1.58	0.38		0.38	0.05

Table S1b Description of upland crop experimental treatments as entered into EX-ACT with calculator-estimated and measured GHG emissions

Treatments				Ex-Ante Carbon Balance Tool												Measured			
				Description					Cropland		Inputs		Estimate (tCO2e ha^{-1} yr^{-1})			Estimate (tCO2e ha^{-1} yr^{-1})			
Country	Crop	Treatment	Reference	Climate	Moisture regime	Regional soil type	Duration: Implementation phase	Duration: Capitalisation phase	Annual systems	Perennial systems	Fertilizers 1	Fertilizers 2	N_2O	CO_2	Total	N_2O	CO_2	Total	SE*
Costa Rica	Coffee	Full sun	Hergoualc'h et al. 2012	Tropical	Wet	Volcanic soils	1	6 (C stock change was measured over 6 years in field study)	None	Perennial remaining perennial	0.18 t/ha N urea	0.07 t/ha N other N fertilizer	1.17	-0.70	0.47	2.01	-5.83	-3.82	1.98
Costa Rica	Coffee	Shade/agroforestry	Hergoualc'h et al. 2012	Tropical	Wet	Volcanic soils	1	6 (C stock change was measured over 6 years in field study)	None	Perennial remaining perennial	0.18 t/ha N urea	0.07 t/ha N other N fertilizer	1.17	-0.70	0.47	2.72	-17.31	-14.59	2.20
Kenya	Napier grass	Manure	Rosenstock et al. forthcoming	Tropical	Dry	HAC soils	1	0	None	Grassland remaining grassland	0.075 t/ha N compost		0.37		0.37	0.23		0.23	
Kenya	Tea	Farm 1	Rosenstock et al. forthcoming	Tropical	Dry	HAC soils	1	0	None	Perennial remaining perennial	0.144 t/ha N other N fertilizer		0.53		0.53	0.22		0.22	
Kenya	Tea	Farm 2	Rosenstock et al. forthcoming	Tropical	Dry	HAC soils	1	0	None	Perennial remaining perennial	0.141 t/ha N other N fertilizer		0.52		0.52	0.11		0.11	
Mexico	Maize	Conventional till, residues removed	Dendooven et al. 2012	Warm temperate	Dry	HAC soils	1	19	No management changes		0.15 t/ha N urea		2.03	0.00	2.03	0.39	0.00	0.39	
Mexico	Maize	Conventional tillage, residues retained	Dendooven et al. 2013	Warm temperate	Dry	HAC soils	1	19	Improved agronomic practices		0.15 t/ha N urea		2.14	-0.94	1.20	0.46	-0.32	0.14	
Mexico	Maize	Zero tillage, residues removed	Dendooven et al. 2014	Warm temperate	Dry	HAC soils	1	19	No till/residues management		0.15 t/ha N urea		2.01	-1.71	0.30	0.33	0.34	0.66	
Mexico	Maize	Zero tillage, residues retained	Dendooven et al. 2015	Warm temperate	Dry	HAC soils	1	19	Improved agronomic practices and no till/residues management		0.15 t/ha N urea		2.20	-3.07	-0.87	0.44	-5.11	-4.68	
Zimbabwe	Maize	No inputs	Nyamadzawo et al. 2014	Tropical	Dry	Sandy soils	1	0	Annual remaining annual; no management changes							0.10		0.10	0.01
Zimbabwe	Maize	Low mineral N	Nyamadzawo et al. 2014	Tropical	Dry	Sandy soils	1	0	Annual remaining annual; no management changes		0.06 t/ha N other N fertilizer		0.28		0.28	0.15		0.15	0.02
Zimbabwe	Maize	High mineral N	Nyamadzawo et al. 2014	Tropical	Dry	Sandy soils	1	0	Annual remaining annual; no management changes		0.12 t/ha N other N fertilizer		0.56		0.56	0.12		0.12	0.01
Zimbabwe	Maize	Mixed fertilization	Nyamadzawo et al. 2014	Tropical	Dry	Sandy soils	1	0	Annual remaining annual; no management changes		0.06 t/ha N other N fertilizer	0.06 t/ha N compost	0.56		0.56	0.10		0.10	0.01
Zimbabwe	Maize	Low organic N	Nyamadzawo et al. 2014	Tropical	Dry	Sandy soils	1	0	Annual remaining annual; no management changes		0.06 t/ha N compost		0.28		0.28	0.08		0.08	0.02
Zimbabwe	Maize	High organic N	Nyamadzawo et al. 2014	Tropical	Dry	Sandy soils	1	0	Annual remaining annual; no management changes		0.12 t/ha N compost		0.55		0.55	0.08		0.08	0.02
Kenya	Vegetables	Manure and mineral fertilizer	Rosenstock et al. forthcoming	Tropical	Moist	LAC soils	1	0	Annual remaining annual; no management changes		0.101 t/ha N other N fertilizer	0.014 t/ha N compost	0.56		0.56	0.26		0.26	
Kenya	Potatoes	Manure	Rosenstock et al. forthcoming	Tropical	Moist	LAC soils	1	0	Annual remaining annual; no management changes		0.024 t/ha N compost		0.12		0.12	0.26		0.26	
Tanzania	Maize	Farmer practice (no inputs)	Rosenstock et al. forthcoming	Tropical	Moist	LAC soils	1	0	Annual remaining annual; no management changes							0.10		0.10	0.61
Tanzania	Maize	Conventional tillag, no inputs	Kimaro et al. 2015	Tropical	Moist	LAC soils	1	0	Annual remaining annual; no management changes							0.08		0.08	0.02
Tanzania	Maize	No-till, leguminous intercrop, residue mulch	Kimaro et al. 2015	Tropical	Moist	LAC soils	1	0	Annual remaining annual; no management changes							0.08		0.08	0.05
Tanzania	Maize	No-till, mineral fertilizer, residue mulch	Kimaro et al. 2015	Tropical	Moist	LAC soils	1	0	Annual remaining annual; no management changes		0.1 t/ha N urea		0.48		0.48	0.10		0.10	0.06
Tanzania	Maize	Heavy mulching in rows	Kimaro et al. 2015	Tropical	Moist	LAC soils	1	0	Annual remaining annual; no management changes							0.07		0.07	0.06
Tanzania	Maize	No-till, residue mulch, interplanting of leguminous trees	Kimaro et al. 2015	Tropical	Moist	LAC soils	1	0	Annual remaining annual; no management changes							0.10		0.10	0.07
Tanzania	Cassava	No inputs	Rosenstock et al. forthcoming	Tropical	Moist	LAC soils	1	0	Annual remaining annual; no management changes							0.13		0.13	0.22
Kenya	Maize	No N	Hickman et al. 2014	Tropical	Dry	HAC soils	1	0	Annual remaining annual; no management changes							0.33		0.33	0.04
Kenya	Maize	50 kg/ha N	Hickman et al. 2014	Tropical	Dry	HAC soils	1	0	Annual remaining annual; no management changes		16.70 kg/ha N other N fertilizer	33.3 kg/ha N urea	0.12		0.12	0.31		0.31	0.05
Kenya	Maize	75 kg/ha N	Hickman et al. 2014	Tropical	Dry	HAC soils	1	0	Annual remaining annual; no management changes		25.00 kg/ha N other N fertilizer	50 kg/ha N urea	0.18		0.18	0.30		0.30	0.05
Kenya	Maize	100 kg/ha N	Hickman et al. 2014	Tropical	Dry	HAC soils	1	0	Annual remaining annual; no management changes		33.30 kg/ha N other N fertilizer	66.7 kg/ha N urea	0.23		0.23	0.29		0.29	0.04
Kenya	Maize	200 kg/ha N	Hickman et al. 2014	Tropical	Dry	HAC soils	1	0	Annual remaining annual; no management changes		66.70 kg/ha N other N fertilizer	133.3 kg/ha N urea	0.47		0.47	0.38		0.38	0.05

Table S2a Description of rice crop experimental treatments as entered into the CFT with calculator-estimated and measured GHG emissions

Treatments				Cool Farm Tool																			Measured			
				Farm Settings		General	Growing area: Soil characteristics					Field treatment								Estimate (tCO2e ha^{-1} yr^{-1})			Estimate (tCO2e ha^{-1} yr^{-1})			
Country	Water treatment	Input treatment	Reference	Climate	Avg temp (°C)	Crop type	Texture	% SOM	Moisture	Drainage	pH	Fertilizer 1	Fertilizer 2	Fertilizer 3	Residue	Rice straw treatment	Days under cultivation	Water mgmt before cultivation	Water mgmt during cultivation	N_2O	CH_4	Total	N_2O	CH_4	Total	SE*
China	Continuous flooding	Mineral fertilizer	Qin et al. 2010	Tropical	25	Rice	Fine	2.63	Moist	Poor	5.5 < pH <= 7.3	100 kg/ha N urea broadcast			n/a	Exported off farm	122	Flooded pre-season	Continuously flooded	0.12	7.93	8.05	0.01	2.63	2.65	0.01
China	Midseason drainage	Mineral fertilizer	Qin et al. 2010	Tropical	25	Rice	Fine	2.63	Moist	Poor	5.5 < pH <= 7.3	100 kg/ha N urea broadcast			n/a	Exported off farm	122	Not flooded pre-season (for < 180	Intermittently flooded	0.12	2.77	2.89	0.27	1.24	1.50	0.03
China	Midseason drainage and moist end of season	Mineral fertilizer	Qin et al. 2010	Tropical	25	Rice	Fine	2.63	Moist	Poor	5.5 < pH <= 7.4	100 kg/ha N urea broadcast			n/a	Exported off farm	122	Not flooded pre-season (for < 180 days)	Intermittently flooded-multiple aeration	0.12	2.46	2.58	0.39	0.92	1.31	0.11
China	Continuous flooding	Compost	Qin et al. 2010	Tropical	25	Rice	Fine	2.63	Moist	Poor	5.5 < pH <= 7.5	40 kg/ha N compost, broadcast	60 kg/ha N compost, incorporated		n/a	Exported off farm	122	Flooded pre-season	Continuously flooded	0.09	9.41	9.50	0.05	3.17	3.22	0.61
China	Midseason drainage	Compost	Qin et al. 2010	Tropical	25	Rice	Fine	2.63	Moist	Poor	5.5 < pH <= 7.6	40 kg/ha N compost, broadcast	60 kg/ha N compost, incorporated		n/a	Exported off farm	122	Not flooded pre-season (for < 180	Intermittently flooded	0.09	2.97	3.06	0.09	1.53	1.62	0.08
China	Midseason drainage and moist end of season	Compost	Qin et al. 2010	Tropical	25	Rice	Fine	2.63	Moist	Poor	5.5 < pH <= 7.7	40 kg/ha N compost, broadcast	60 kg/ha N compost, incorporated		n/a	Exported off farm	122	Not flooded pre-season (for < 180 days)	Intermittently flooded-multiple aeration	0.09	2.58	2.67	0.12	1.25	1.36	0.21
Cambodia	Continuous flooding	No inputs	Ly et al. 2013	Tropical	27	Rice	Coarse	0.49	Moist	Good	pH <= 5.5				1.5 t/ha	Left on field; incorporated shortly before cultivation	78	Flooded pre-season	Continuously flooded		8.27	8.27		3.88	3.88	0.32
Cambodia	Continuous flooding	Manure	Ly et al. 2013	Tropical	27	Rice	Coarse	0.49	Moist	Good	pH <= 5.5	5.5 t/ha cattle farmyard manure, incorporated			1.5 t/ha	Left on field; incorporated shortly before cultivation	78	Flooded pre-season	Continuously flooded		9.69	9.69		5.69	5.69	0.72
Cambodia	Continuous flooding	Mixed fertilization	Ly et al. 2013	Tropical	27	Rice	Coarse	0.49	Moist	Good	pH <= 5.5	5.5 t/ha cattle farmyard manure, incorporated	50 kg/ha urea, broadcast	100 kg/ha compound NPK, broadcast	1.5 t/ha	Left on field; incorporated shortly before cultivation	78	Flooded pre-season	Continuously flooded		9.69	9.69		7.06	7.06	1.17
Cambodia	Continuous flooding	Mineral fertilizer	Ly et al. 2013	Tropical	27	Rice	Coarse	0.49	Moist	Good	pH <= 5.5	50 kg/ha urea, broadcast	100 kg/ha compound NPK, broadcast		1.5 t/ha	Left on field; incorporated shortly before cultivation	78	Flooded pre-season	Continuously flooded		8.27	8.27		4.90	4.90	0.94
Cambodia	Multiple aeration	No inputs	Ly et al. 2013	Tropical	27	Rice	Coarse	0.49	Moist	Good	pH <= 5.5				1.5 t/ha	Left on field; incorporated shortly before cultivation	78	Flooded pre-season	Intermittently flooded-multiple aeration		4.30	4.30		3.90	3.90	1.32
Cambodia	Multiple aeration	Manure	Ly et al. 2013	Tropical	27	Rice	Coarse	0.49	Moist	Good	pH <= 5.5	5.5 t/ha cattle farmyard manure, incorporated			1.5 t/ha	Left on field; incorporated shortly before cultivation	78	Flooded pre-season	Intermittently flooded-multiple aeration		5.04	5.04		4.44	4.44	0.95
Cambodia	Multiple aeration	Mixed fertilization	Ly et al. 2013	Tropical	27	Rice	Coarse	0.49	Moist	Good	pH <= 5.5	5.5 t/ha cattle farmyard manure, incorporated	50 kg/ha urea, broadcast	100 kg/ha compound NPK, broadcast	1.5 t/ha	Left on field; incorporated shortly before cultivation	78	Flooded pre-season	Intermittently flooded-multiple aeration		5.04	5.04		5.33	5.33	1.30
Cambodia	Multiple aeration	Mineral fertilizer	Ly et al. 2013	Tropical	27	Rice	Coarse	0.49	Moist	Good	pH <= 5.5	50 kg/ha urea, broadcast	100 kg/ha compound NPK, broadcast		1.5 t/ha	Left on field; incorporated shortly before cultivation	78	Flooded pre-season	Intermittently flooded-multiple aeration		4.30	4.30		4.08	4.08	0.62
Vietnam	Continuous flooding	Mineral fertilizer	Pandey et al. 2014	Tropical	24	Rice	Medium	2.16	Moist	Good	5.5 < pH <= 7.7	100 kg/ha N urea, broadcast			n/a	Exported off farm	84	Not flooded pre-season (for < 180	Continuously flooded	0.05	2.73	2.78	0.09	2.70	2.79	0.17
Vietnam	Continuous flooding	Manure	Pandey et al. 2014	Tropical	24	Rice	Medium	2.16	Moist	Good	5.5 < pH <= 7.7	100 kg/ha N urea, broadcast	.01801 t/ha N pig farmyard manure, broadcast		n/a	Exported off farm	84	Not flooded pre-season (for < 180 days)	Continuously flooded	0.06	3.27	3.33	0.13	8.83	8.96	0.10
Vietnam	Continuous flooding	Compost	Pandey et al. 2014	Tropical	24	Rice	Medium	2.16	Moist	Good	5.5 < pH <= 7.7	100 kg/ha N urea, broadcast	0.02977 t/ha N compost		n/a	Exported off farm	84	Not flooded pre-season (for < 180	Continuously flooded	0.06	2.73	2.79	0.13	6.30	6.43	0.87
Vietnam	Continuous flooding	Biochar	Pandey et al. 2014	Tropical	24	Rice	Medium	2.16	Moist	Good	5.5 < pH <= 7.7	100 kg/ha N urea, broadcast	0.00787 t/ha N compost		n/a	Exported off farm	84	Not flooded pre-season (for < 180	Continuously flooded	0.05	2.73	2.78	0.08	3.50	3.58	0.12
Vietnam	Multiple aeration	Mineral fertilizer	Pandey et al. 2014	Tropical	24	Rice	Medium	2.16	Moist	Good	5.5 < pH <= 7.7	100 kg/ha N urea, broadcast			n/a	Exported off farm	84	Not flooded pre-season (for < 180	Intermittently flooded-multiple aeration	0.05	1.42	1.47	0.22	0.78	1.00	0.13
Vietnam	Multiple aeration	Manure	Pandey et al. 2014	Tropical	24	Rice	Medium	2.16	Moist	Good	5.5 < pH <= 7.7	100 kg/ha N urea, broadcast	.01801 t/ha N pig farmyard manure, broadcast		n/a	Exported off farm	84	Not flooded pre-season (for < 180 days)	Intermittently flooded-multiple aeration	0.06	1.70	1.76	0.29	2.63	2.91	0.17
Vietnam	Multiple aeration	Compost	Pandey et al. 2014	Tropical	24	Rice	Medium	2.16	Moist	Good	5.5 < pH <= 7.7	100 kg/ha N urea, broadcast	0.02977 t/ha N compost		n/a	Exported off farm	84	Not flooded pre-season (for < 180	Intermittently flooded-multiple aeration	0.06	1.42	1.48	0.23	1.98	2.21	0.17
Vietnam	Multiple aeration	Biochar	Pandey et al. 2014	Tropical	24	Rice	Medium	2.16	Moist	Good	5.5 < pH <= 7.7	100 kg/ha N urea, broadcast	0.00787 t/ha N compost		n/a	Exported off farm	84	Not flooded pre-season (for < 180	Intermittently flooded-multiple aeration	0.05	1.42	1.47	0.20	1.15	1.35	0.07

Table S2b Description of rice crop experimental treatments as entered into EX-ACT with calculator-estimated and measured GHG emissions

Treatments				Ex-Ante Carbon Balance Tool															Measured			
				Description					Cropland					Inputs		Estimate (tCO2e ha^{-1} yr^{-1})			Estimate (tCO2e ha^{-1} yr^{-1})			
Country	Water treatment	Input treatment	Reference	Climate	Moisture regime	Regional soil type	Duration: Implementation phase	Duration: Capitalisation phase	Annual systems	Cultivation period (da7s)	Water regime during cultivation	Water regime before cultivation	Organic amendment type	Fertilizers 1	Fertilizers 2	N_2O	CH_4	Total	N_2O	CH_4	Total	SE*
China	Continuous flooding	Mineral fertilizer	Qin et al. 2010	Tropical	Moist	LAC soils	1	0	Flooded rice remaining flooded rice	122	Continuously flooded	Flooded pre-season (> 30 days)		0.1 t/ha N urea		0.62	7.53	8.16	0.01	2.63	2.65	0.01
China	Midseason drainage	Mineral fertilizer	Qin et al. 2010	Tropical	Moist	LAC soils	1	0	Flooded rice remaining flooded rice	122	Intermittently flooded	Not flooded pre-season (< 180 days)		0.1 t/ha N urea		0.62	2.22	2.85	0.27	1.24	1.50	0.03
China	Midseason drainage and moist end of season	Mineral fertilizer	Qin et al. 2010	Tropical	Moist	LAC soils	1	0	Flooded rice remaining flooded rice	122	Intermittently flooded	Not flooded pre-season (< 180 days)		0.1 t/ha N urea		0.62	2.22	2.85	0.39	0.92	1.31	0.11
China	Continuous flooding	Compost	Qin et al. 2010	Tropical	Moist	LAC soils	1	0	Flooded rice remaining flooded rice	122	Continuously flooded	Flooded pre-season (> 30 days)	Compost	0.1 t/ha N compost		0.47	7.53	8.00	0.05	3.17	3.22	0.61
China	Midseason drainage	Compost	Qin et al. 2010	Tropical	Moist	LAC soils	1	0	Flooded rice remaining flooded rice	122	Intermittently flooded	Not flooded pre-season (< 180 days)	Compost	0.1 t/ha N compost		0.47	2.22	2.69	0.09	1.53	1.62	0.08
China	Midseason drainage and moist end of season	Compost	Qin et al. 2010	Tropical	Moist	LAC soils	1	0	Flooded rice remaining flooded rice	122	Intermittently flooded	Not flooded pre-season (< 180 days)	Compost	0.1 t/ha N compost		0.47	2.22	2.69	0.12	1.25	1.36	0.21
Cambodia	Continuous flooding	No inputs	Ly et al. 2013	Tropical	Moist	LAC soils	1	0	Flooded rice remaining flooded rice	78	Continuously flooded	Flooded pre-season (> 30 days)	Straw incorporated shortly before cultivation	n/a (N2O emissions not measured in this study)			14.53	14.53		3.88	3.88	0.32
Cambodia	Continuous flooding	Manure	Ly et al. 2013	Tropical	Moist	LAC soils	1	0	Flooded rice remaining flooded rice	78	Continuously flooded	Flooded pre-season (> 30 days)	Manure	n/a (N2O emissions not measured in this study)			6.75	6.75		5.69	5.69	0.72
Cambodia	Continuous flooding	Mixed fertilization	Ly et al. 2013	Tropical	Moist	LAC soils	1	0	Flooded rice remaining flooded rice	78	Continuously flooded	Flooded pre-season (> 30 days)	Manure	n/a (N2O emissions not measured in this study)			6.75	6.75		7.06	7.06	1.17
Cambodia	Continuous flooding	Mineral fertilizer	Ly et al. 2013	Tropical	Moist	LAC soils	1	0	Flooded rice remaining flooded rice	78	Continuously flooded	Flooded pre-season (> 30 days)	Straw incorporated shortly before cultivation	n/a (N2O emissions not measured in this study)			6.75	6.75		4.90	4.90	0.94
Cambodia	Multiple aeration	No inputs	Ly et al. 2013	Tropical	Moist	LAC soils	1	0	Flooded rice remaining flooded rice	78	Intermittently flooded	Flooded pre-season (> 30 days)	Straw incorporated shortly before cultivation	n/a (N2O emissions not measured in this study)			8.14	8.14		3.90	3.90	1.32
Cambodia	Multiple aeration	Manure	Ly et al. 2013	Tropical	Moist	LAC soils	1	0	Flooded rice remaining flooded rice	78	Intermittently flooded	Flooded pre-season (> 30 days)	Manure	n/a (N2O emissions not measured in this study)			3.78	3.78		4.44	4.44	0.95
Cambodia	Multiple aeration	Mixed fertilization	Ly et al. 2013	Tropical	Moist	LAC soils	1	0	Flooded rice remaining flooded rice	78	Intermittently flooded	Flooded pre-season (> 30 days)	Manure	n/a (N2O emissions not measured in this study)			3.78	3.78		5.33	5.33	1.30
Cambodia	Multiple aeration	Mineral fertilizer	Ly et al. 2013	Tropical	Moist	LAC soils	1	0	Flooded rice remaining flooded rice	78	Intermittently flooded	Flooded pre-season (> 30 days)	Straw incorporated shortly before cultivation	n/a (N2O emissions not measured in this study)			3.78	3.78		4.08	4.08	0.62
Vietnam	Continuous flooding	Mineral fertilizer	Pandey et al. 2014	Tropical	Moist	LAC soils	1	0	Flooded rice remaining flooded rice	84	Continuously flooded	Not flooded pre-season (< 180 days)		0.1 t/ha N urea		0.49	2.29	2.78	0.09	2.70	2.79	0.17
Vietnam	Continuous flooding	Manure	Pandey et al. 2014	Tropical	Moist	LAC soils	1	0	Flooded rice remaining flooded rice	84	Continuously flooded	Not flooded pre-season (< 180 days)	Manure	0.1 t/ha N urea	0.018 t/ha N compost	0.57	3.21	3.79	0.13	8.83	8.96	0.10
Vietnam	Continuous flooding	Compost	Pandey et al. 2014	Tropical	Moist	LAC soils	1	0	Flooded rice remaining flooded rice	84	Continuously flooded	Not flooded pre-season (< 180 days)	Compost	0.1 t/ha N urea	0.03 t/ha N compost	0.15	2.65	2.79	0.13	6.30	6.43	0.87
Vietnam	Continuous flooding	Biochar	Pandey et al. 2014	Tropical	Moist	LAC soils	1	0	Flooded rice remaining flooded rice	84	Continuously flooded	Not flooded pre-season (< 180 days)	Compost	0.1 t/ha N urea	.008 t/ha compost	0.04	2.65	2.68	0.08	3.50	3.58	0.12
Vietnam	Multiple aeration	Mineral fertilizer	Pandey et al. 2014	Tropical	Moist	LAC soils	1	0	Flooded rice remaining flooded rice	84	Intermittently flooded	Not flooded pre-season (< 180 days)		0.1 t/ha N urea		0.49	1.28	1.77	0.22	0.78	1.00	0.13
Vietnam	Multiple aeration	Manure	Pandey et al. 2014	Tropical	Moist	LAC soils	1	0	Flooded rice remaining flooded rice	84	Intermittently flooded	Not flooded pre-season (< 180 days)	Manure	0.1 t/ha N urea	0.018 t/ha N compost	0.57	1.80	2.37	0.29	2.63	2.91	0.17
Vietnam	Multiple aeration	Compost	Pandey et al. 2014	Tropical	Moist	LAC soils	1	0	Flooded rice remaining flooded rice	84	Intermittently flooded	Not flooded pre-season (< 180 days)	Compost	0.1 t/ha N urea	0.03 t/ha N compost	0.15	1.48	1.63	0.23	1.98	2.21	0.17
Vietnam	Multiple aeration	Biochar	Pandey et al. 2014	Tropical	Moist	LAC soils	1	0	Flooded rice remaining flooded rice	84	Intermittently flooded	Not flooded pre-season (< 180 days)	Compost	0.1 t/ha N urea	0.008 t/ha compost	0.04	1.48	1.52	0.20	1.15	1.35	0.07
